# Diagnosing and treating epiphora in the 21^st^ century

**DOI:** 10.5935/0004-2749.2024-0099

**Published:** 2024-08-30

**Authors:** Patricia Santello Akaishi, Silvana Artioli Schellini

**Affiliations:** 1 Departamento de Oftalmologia e Otorrinolaringologia, Faculdade de Medicina de Ribeirão Preto, Universidade de São Paulo, São Paulo, SP, Brazil; 2 Departamento de Anestesiologia e Especialidades Cirúrgicas, Faculdade de Medicina de Botucatu, Universidade Estadual Paulista “Júlio de Mesquita Filho”, Botucatu, SP, Brazil

Dear Editor,

The term epiphora refers to excessive tearing due to insufficient tear drainage. It is a common complaint in ophthalmological offices and the daily practice of oculoplastic surgeons. Epiphora can cause discomfort and interfere with patients’ activities. Although tear obstruction is a common cause of epiphora, less than half of the patients with epiphora have obstruction of the lacrimal drainage system (LDS)^(1)^. Non-obstructive epiphora may be attributed to eyelid malposition, inflammation of the eyelid margin such as meibomitis and/or blepharitis, keratinization, or absence of lacrimal puncta. These conditions can be easily diagnosed during routine ophthalmological consultations. Obstruction of the LDS can affect the proximal/upper part (puncti and canaliculi) and the distal/lower part (lacrimal sac and nasolacrimal duct). Thus, proper diagnosis is mandatory for its management.

Until the mid-20^th^ century, most of the LDS knowledge came from anatomical studies of cadavers. As imaging diagnosis became more accessible, with better resolution and quality, anatomical information regarding tear drainage physiology has been updated.

The first observations of *in vivo* tear drainage were made at the end of the 20^th^ century, when nasal endoscopy was used to visualize the lacrimal probe in the inferior meatus in patients with congenital nasolacrimal duct obstruction (CNLDO)^(2)^. They verified that 36% of children have a submucosal duct in the nasal cavity. Several subsequent publications have confirmed the benefit of endoscopy-assisted probing, adding to the knowledge on drainage ostium anomalies and their treatment^(3)^.

Another new modality for evaluating the LDS is dacryoendoscopy, which is performed with a microendoscope and a probing system equipped with optical fibers that allow the examiner to guide the probe throughout the entire intraluminal drainage system, from the canaliculus to the Hasner’s valve. This optical device enables the precise identification of any site of obstruction^(4)^.

The more accurate the anatomical diagnosis is, the more effective and safer the treatment becomes. Lacrimal endoscopy has altered the treatment of tear obstructions. The cost of the technology is offset by the greater treatment effectiveness, lower procedure failure rates, and lesser re-exposure to anesthetics, especially general anesthesia. Furthermore, the ability to directly visualize the lacrimal drainage system reduces the risk of iatrogenic injuries^(5)^.

Given the latest developments, there are numerous opportunities to improve traditional techniques that have been considered the gold standard of epiphora management. Time-honored surgeries for lacrimal issues, such as external dacryocystorhinostomy, must coexist with modern endonasal techniques to broaden the surgeon’s repertoire and solve lacrimal disorders. Endoscopic guidance has revolutionized lacrimal surgeries and deepened our understanding of the anatomy, physiology, and pathology of lacrimal disorders.

In the 21^st^ century, endoscopy should be incorporated into the treatment of obstructive lacrimal disorders. Thus, lacrimal endoscopy should be introduced from the first year of oculoplastic fellowship training and should be a goal of all oculoplastic teaching services.
